# Lithium dipotassium citrate monohydrate, LiK_2_C_6_H_5_O_7_(H_2_O)

**DOI:** 10.1107/S2056989021003339

**Published:** 2021-04-09

**Authors:** Andrew J. Cigler, James A. Kaduk

**Affiliations:** a Department of Chemistry, North Central College, 131 S. Loomis St., Naperville IL 60540, USA

**Keywords:** powder diffraction, density functional theory, citrate lithium, potassium

## Abstract

The crystal structure of dilithium potassium citrate monohydrate, Li^+^·2K^+^·C_6_H_5_O_7_^3−^·H_2_O or LiK_2_C_6_H_5_O_7_·H_2_O, has been solved by direct methods and refined against laboratory X-ray powder diffraction data, and optimized using density functional techniques. The complete citrate trianion is generated by a crystallographic mirror plane, with two C and three O atoms lying on the reflecting plane, and chelates to three different K cations. The KO_8_ and LiO_4_ coordination polyhedra share edges and corners to form layers lying parallel to the *ac* plane. An intra­molecular O—H⋯O hydrogen bond occurs between the hydroxyl group and the central carboxyl­ate group of the citrate anion as well as a charge-assisted inter­molecular O—H⋯O link between the water mol­ecule and the terminal carboxyl­ate group. There is also a weak C—H⋯O hydrogen bond.

## Abstract 2

The crystal structure of lithium dipotassium citrate has been solved and refined using laboratory X-ray powder diffraction data, and optimized using density functional techniques.

## Chemical context   

A systematic study of the crystal structures of Group 1 (alkali metal) citrate salts has been reported in Rammohan & Kaduk (2018 ▸). The study was extended to lithium hydrogen citrates in Cigler & Kaduk (2018 ▸), to sodium hydrogen citrates in Cigler & Kaduk (2019*a* ▸), to sodium dirubidium citrates in Cigler & Kaduk (2019*b* ▸) and to dilithium potassium citrate (Cigler & Kaduk, 2019*c* ▸). We now report the synthesis and structure of the title compound, LiK_2_C_6_H_5_O_7_(H_2_O), which represents a further extension to lithium dipotassium citrates.
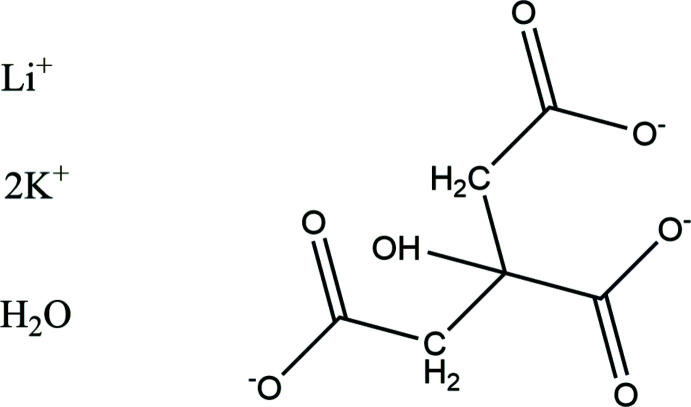


## Structural commentary   

**Figure 1:**
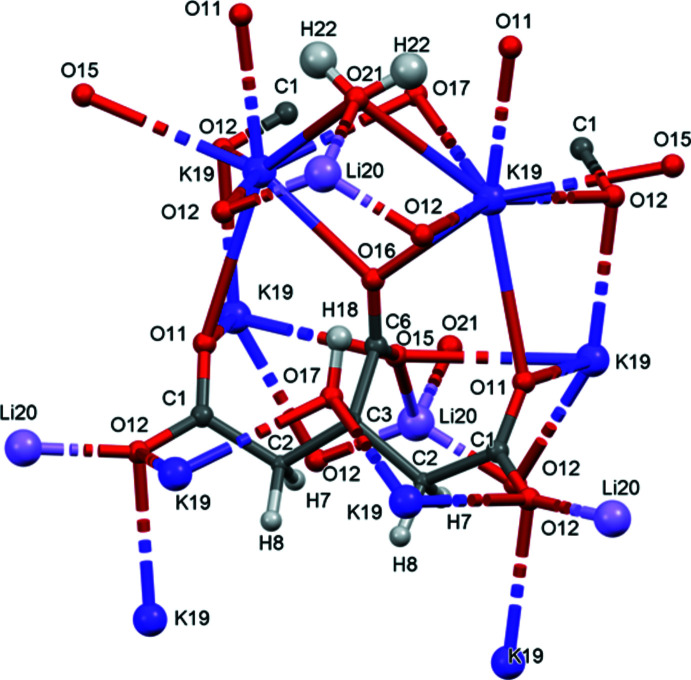
The crystal structure of LiK_2_C_6_H_5_O_7_(H_2_O) with the atom numbering and 50% probability spheroids.

**Figure 2:**
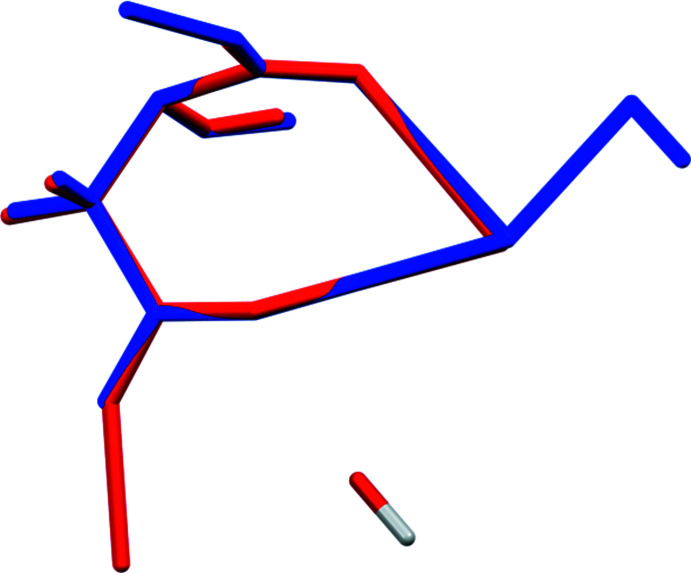
Comparison of the refined and optimized structures of LiK_2_C_6_H_5_O_7_(H_2_O). The refined structure is in red, and the DFT-optimized structure is in blue.

The structure of LiK_2_C_6_H_5_O_7_(H_2_O) was solved and refined from powder data and optimized by density functional theory (DFT) calculations (see *Experimental* section) and is illustrated in Fig. 1 ▸. The root-mean-square Cartesian displacement of the hon-hydrogen atoms in the refined and optimized structures is 0.047 Å (Fig. 2 ▸). The excellent agreement between the structures is evidence that the experimental structure is correct (van de Streek & Neumann, 2014 ▸). All of the citrate bond distances, bond angles, and torsion angles fall within the normal ranges indicated by a *Mercury* Mogul geometry check (Macrae *et al.*, 2020 ▸). The citrate anion occurs in the *trans,trans*-conformation (about C2—C3 and the symmetry-related atoms), which is one of the two low-energy conformations of an isolated citrate anion (Rammohan & Kaduk, 2018 ▸). Since C3, the central C6/O15/O16 carboxyl­ate group and the O17—H18 hy­droxy group lie on the mirror plane, they exhibit the normal planar arrangement. The Mulliken overlap populations indicate that both the Li—O and K—O bonds have some covalent character, but that the Li—O bonds are more covalent.

The C_6_H_5_O_7_^3–^ citrate anion doubly chelates to three different K19 ions though O11/O16, O11/O15 and O12/O17. Each citrate oxygen atom bridges multiple metal atoms. K19 is eight-coordinate (irregular), with a bond-valence sum (in valence units) of 1.04 and Li20 (site symmetry *m*) is tetra­hedral with a bond-valence sum of 1.10. Atom O21 of the water mol­ecule of crystallization also lies on a (100) mirror plane.

The Bravais–Friedel–Donnay–Harker (Bravais, 1866 ▸; Friedel, 1907 ▸; Donnay & Harker, 1937 ▸) method suggests that we might expect a blocky morphology for lithium dipotassium citrate monohydrate. A 2nd order spherical harmonic preferred orientation model was included in the refinement; the texture index was 1.000, indicating that preferred orientation was not present for this rotated capillary specimen.

## Supra­molecular features   

**Figure 3:**
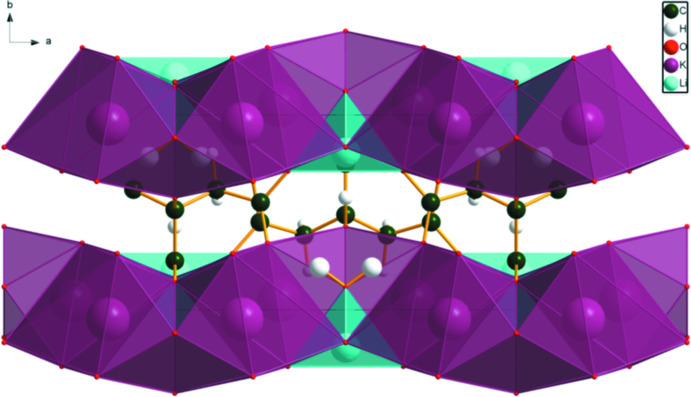
The crystal structure of LiK_2_C_6_H_5_O_7_(H_2_O), viewed down the *c* axis.

The KO_8_ and LiO_4_ coordination polyhedra share edges and corners to form layers lying parallel to the *ac* plane (Fig. 3 ▸). The only traditional hydrogen bonds are an intra­molecular O17—H18⋯O16 inter­action between the hydroxyl group and the central carboxyl­ate group (Table 1 ▸), and a charge-assisted hydrogen bond between the water mol­ecule O21—H22 and O11. By the correlation of Rammohan & Kaduk (2018 ▸), these hydrogen bonds contribute 13.2 and 13.4 kcal mol^−1^, respectively, to the crystal energy. There is also a weak C2—H7⋯O11 hydrogen bond (Table 1 ▸).

## Database survey   

Details of the comprehensive literature search for citrate structures are presented in Rammohan & Kaduk (2018 ▸). A reduced cell search in the Cambridge Structural Database (Groom *et al.*, 2016 ▸) yielded two hits, but no citrate structures. A few weak unindexed peaks were identified as 2.0 wt% dilithium potassium citrate (Cigler & Kaduk, 2019*c* ▸).

## Synthesis and crystallization   

Masses of 0.3777 g of Li_2_CO_3_ (5.00 mmol, Sigma-Aldrich) and 1.3851 g of K_2_CO_3_ (10.0 mmol, Sigma-Aldrich) were added to a solution of 2.0325 g of citric acid (10.0 mmol, Sigma–Aldrich) monohydrate in 15 ml of water. After the fizzing subsided, the clear solution was dried first at 450 K to yield a sticky solid. The solid was heated at 477 K to yield a white foam. Further heating at 505 K yielded additional expansion of the foam, and slight discoloration. This foam was amorphous. Storage of the foam under ambient conditions yielded a puddle. Heating this puddle to 394 K yielded a glassy solid. Adding two drops of water to this solid yielded a paste, which yielded the title compound as a crystalline white powder after heating to 394 K for 15 min.

## Refinement   

**Figure 4:**
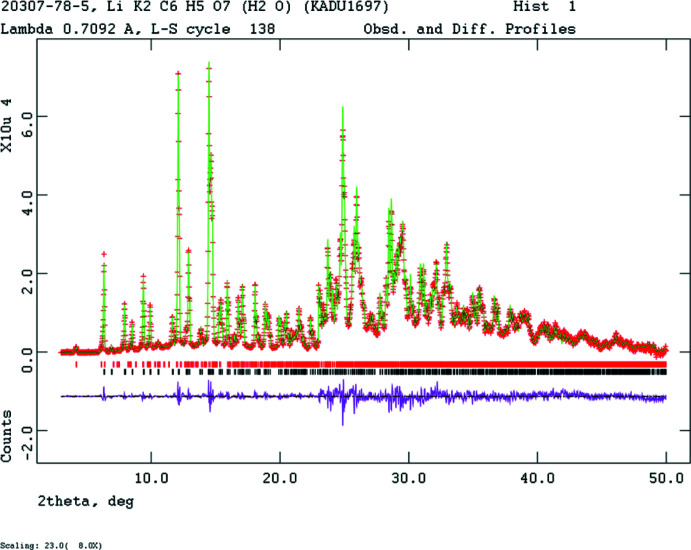
Observed, calculated, and difference patterns of LiK_2_C_6_H_5_O_7_(H_2_O). The red crosses represent the observed data points, the green solid line the calculated pattern, and the magenta line the difference (observed - calculated) pattern. The vertical scale is multiplied by a factor of 8 above 23° 2θ.

The pattern of LiK_2_C_6_H_5_O_7_(H_2_O) was indexed using *Jade* 9.8 (MDI, 2017 ▸). *EXPO2014* (Altomare *et al.*, 2013 ▸) suggested the space group *Pmn2_1_*, which was confirmed by successful solution and refinement of the structure. The structure of LiK_2_C_6_H_5_O_7_(H_2_O) was solved by direct methods as implemented in *EXPO2014* (Altomare *et al.*, 2013 ▸), which located all the non-hydrogen atoms including the lithium atom. The positions of H7 and H8 were calculated using *Materials Studio* (Dassault, 2018 ▸). The position of the active hydrogen atom H18 was deduced from the potential intra­molecular hydrogen-bonding pattern, and the position of H22 was deduced from the hydrogen-bonding pattern. Pseudo-Voigt profile coefficients were as parameterized in Thompson *et al.* (1987 ▸) and the asymmetry correction of Finger *et al.* (1994 ▸) was applied and the microstrain broadening model of Stephens (1999 ▸). The hydrogen atoms were included in fixed positions, which were re-calculated during the course of the refinement using *Materials Studio*. Crystal data, data collection and structure refinement (Fig. 4 ▸) details are summarized in Table 2 ▸. The *U*_iso_ values for C2 and C3 were constrained to be equal, and those of H7 and H8 were constrained to be 1.3× that of these carbon atoms. The *U*_iso_ of C1, C5, C6 and the oxygen atoms were constrained to be equal, and that of H18 was constrained to be 1.3× this value. The background was modeled by a three-term shifted Chebyshev polynomial. A ten-term diffuse scattering function was used to describe the scattering from the capillary and any amorphous material. The structure of dilithium potassium citrate, Li_2_KC_6_H_5_O_7_ (Cigler & Kaduk, 2019*c* ▸), was included as a second phase in the Rietveld refinement but its atomic positional and displacement parameters were not refined.

A density functional geometry optimization was carried out using *CRYSTAL14* (Dovesi *et al.*, 2014 ▸). The basis sets for the H, C, N, and O atoms were those of Gatti *et al.* (1994 ▸), and the basis set for K was that of Peintinger *et al.* (2013 ▸). The calculation was run on eight 2.1 GHz Xeon cores (each with 6 Gb RAM) of a 304-core Dell Linux cluster at IIT, using 8 *k*-points and the B3LYP functional, and took two hours.

## Supplementary Material

Crystal structure: contains datablock(s) KADU1697_publ, kadu1697_DFT, KADU1697_overall, KADU1697_phase_1, KADU1697_phase_2, KADU1697_p_01. DOI: 10.1107/S2056989021003339/hb7968sup1.cif

Supporting information file. DOI: 10.1107/S2056989021003339/hb7968KADU1697_phase_1sup2.cml

CCDC references: 2074045, 2074046, 2074047

Additional supporting information:  crystallographic information; 3D view; checkCIF report

## Figures and Tables

**Table 1:** Hydrogen-bond geometry (Å, °) for kadu1697_DFT[Chem scheme1]

*D*—H⋯*A*	*D*—H	H⋯*A*	*D*⋯*A*	*D*—H⋯*A*
O21—H22⋯O11	0.98	1.73	2.687	164
O17—H18⋯O16	0.98	1.90	2.581	124
C2—H7⋯O11^i^	1.09	2.47	3.396	142

Symmetry code: (i) -x+{\script{1\over 2}}, -y+1, z-{\script{1\over 2}}.

**Table 2:** Experimental details

	KADU1697_phase_1
Crystal data	
Chemical formula	Li^+^·2K^+^·C_6_H_5_O_7_^3−^·H_2_O
*M*_r_	292.25
Crystal system, space group	Orthorhombic, *P* *m* *n*2_1_
Temperature (K)	300
*a*, *b*, *c* (Å)	10.24878 (19), 5.86577 (14), 8.19290 (16)
*V* (Å^3^)	492.53 (1)
*Z*	2
Radiation type	*K*α_1_, *K*α_2_, λ = 0.709237, 0.713647 Å
Specimen shape, size (mm)	Cylinder, 12 × 0.7

Data collection	
Diffractometer	PANalytical Empyrean
Specimen mounting	Glass capillary
Data collection mode	Transmission
Scan method	Step
2θ values (°)	2θ_min_ = 1.008, 2θ_max_ = 49.988, 2θ_step_ = 0.017

Refinement	
*R* factors and goodness of fit	*R*_p_ = 0.034, *R*_wp_ = 0.044, *R*_exp_ = 0.015, *R*(*F* ^2^) = 0.04860, χ^2^ = 8.940
No. of parameters	56
No. of restraints	14
(Δ/σ)_max_	0.49

Computer programs: *EXPO2014* (Altomare *et al.*, 2013 ▸), *GSAS* (Toby & Von Dreele, 2013 ▸), *Mercury* (Macrae *et al.*, 2020 ▸), *DIAMOND* (Crystal Impact, 2015 ▸), and *publCIF* (Westrip, 2010 ▸).

## supplementary crystallographic information

### Lithium dipotassium citrate monohydrate (KADU1697_phase_1) Crystal data

**Table:**

Li^+^·2K^+^·C_6_H_5_O_7_^3^^−^·H_2_O	*c* = 8.19290 (16) Å
*M**_r_* = 292.25	*V* = 492.53 (1) Å^3^
Orthorhombic, *P**m**n*2_1_	*Z* = 2
Hall symbol: P 2ac -2	*D*_x_ = 1.971 Mg m^−^^3^
*a* = 10.24878 (19) Å	*T* = 300 K
*b* = 5.86577 (14) Å	

### Lithium dipotassium citrate monohydrate (KADU1697_phase_1) Fractional atomic coordinates and isotropic or equivalent isotropic displacement parameters (Å^2^)

**Table:**

	*x*	*y*	*z*	*U*_iso_*/*U*_eq_	
C1	0.2488 (7)	0.5454 (12)	0.9115 (17)	0.0155 (10)*	
C2	0.1204 (5)	0.6153 (14)	0.8359 (11)	0.010 (2)*	
C3	0.0	0.5097 (15)	0.9185	0.010 (2)*	
C6	0.0	0.2480 (14)	0.8941 (14)	0.0155 (10)*	
H7	0.125	0.5784	0.7037	0.013 (3)*	
H8	0.1091	0.8242	0.8467	0.013 (3)*	
O11	0.2709 (4)	0.3382 (8)	0.9343 (12)	0.0155 (10)*	
O12	0.3320 (4)	0.6995 (7)	0.9364 (12)	0.0155 (10)*	
O15	0.0	0.1757 (12)	0.7512 (12)	0.0155 (10)*	
O16	0.0	0.1310 (11)	1.0247 (12)	0.0155 (10)*	
O17	0.0	0.5645 (12)	1.0886 (8)	0.0155 (10)*	
H18	0.0	0.4231	1.1176	0.0201 (13)*	
K19	0.20108 (18)	−0.0384 (3)	1.1763 (10)	0.0333 (7)*	
Li20	0.0	0.213 (4)	1.526 (3)	0.04*	
O21	0.5	0.1259 (10)	0.9430 (16)	0.018 (3)*	
H22	0.4268	0.1937	0.9402	0.04*	

### Lithium dipotassium citrate monohydrate (KADU1697_phase_1) Geometric parameters (Å, º)

**Table:**

C1—C2	1.510 (3)	O16—C6	1.271 (6)
C1—O11	1.250 (5)	O16—K19	2.604 (4)
C1—O12	1.260 (6)	O16—K19^i^	2.604 (4)
C2—C1	1.510 (3)	O17—C3	1.430 (7)
C2—C3	1.538 (3)	O17—H18	0.863 (7)
C2—H7	1.105 (10)	O17—K19^iii^	3.192 (5)
C2—H8	1.234 (8)	O17—K19^viii^	3.192 (5)
C3—C2	1.538 (3)	H18—O17	0.863 (7)
C3—C2^i^	1.538 (3)	K19—H8^ix^	2.704 (4)
C3—C6	1.548 (3)	K19—O11	3.053 (5)
C3—O17	1.430 (7)	K19—O11^x^	2.765 (5)
C6—C3	1.548 (3)	K19—O12^xi^	2.833 (5)
C6—O15	1.245 (7)	K19—O12^ix^	2.934 (5)
C6—O16	1.271 (6)	K19—O15^xii^	3.226 (3)
H7—C2	1.105 (10)	K19—O16	2.604 (4)
H8—C2	1.234 (8)	K19—O17^xi^	3.192 (5)
O11—C1	1.250 (5)	K19—O21^xiii^	3.047 (7)
O11—K19	3.053 (5)	Li20—O12^xiv^	1.941 (12)
O11—K19^ii^	2.765 (5)	Li20—O12^ix^	1.941 (12)
O12—C1	1.260 (6)	Li20—O15^xv^	1.86 (2)
O12—K19^iii^	2.833 (5)	Li20—O21^xiii^	2.11 (2)
O12—K19^iv^	2.934 (5)	O21—K19^xvi^	3.047 (7)
O12—Li20^v^	1.941 (12)	O21—K19^ii^	3.047 (7)
O15—C6	1.245 (7)	O21—Li20^xvi^	2.11 (2)
O15—K19^vi^	3.226 (3)	O21—H22^xvii^	0.908 (5)
O15—K19^ii^	3.226 (3)	O21—H22^ix^	0.908 (5)
O15—Li20^vii^	1.86 (2)	H22—O21^xviii^	0.908 (5)
			
C2—C1—O11	118.9 (6)	O11—K19—O12^xi^	80.28 (12)
C2—C1—O12	117.5 (6)	O11—K19—O12^ix^	90.49 (12)
O11—C1—O12	123.4 (6)	O11—K19—O15^xii^	94.67 (13)
C1—C2—C3	114.1 (5)	O11—K19—O16	66.39 (15)
C1—C2—H7	108.2 (6)	O11—K19—O17^xi^	122.22 (14)
C1—C2—H8	108.8 (6)	O11—K19—O21^xiii^	138.24 (16)
C3—C2—H7	112.7 (6)	O11^x^—K19—O12^xi^	97.83 (17)
C3—C2—H8	107.0 (6)	O11^x^—K19—O12^ix^	83.53 (13)
H7—C2—H8	105.6 (5)	O11^x^—K19—O15^xii^	66.25 (14)
C2—C3—C2^i^	106.8 (6)	O11^x^—K19—O16	133.62 (19)
C2—C3—C6	110.0 (5)	O11^x^—K19—O17^xi^	77.00 (13)
C2—C3—O17	109.8 (5)	O11^x^—K19—O21^xiii^	54.18 (14)
C2^i^—C3—C6	110.0 (5)	O12^xi^—K19—O12^ix^	157.86 (7)
C2^i^—C3—O17	109.8 (5)	O12^xi^—K19—O15^xii^	63.05 (15)
C6—C3—O17	110.4 (7)	O12^xi^—K19—O16	104.54 (18)
C3—C6—O15	117.3 (8)	O12^xi^—K19—O17^xi^	75.75 (14)
C3—C6—O16	115.3 (8)	O12^xi^—K19—O21^xiii^	136.58 (15)
O15—C6—O16	127.4 (9)	O12^ix^—K19—O15^xii^	98.08 (14)
C1—O11—K19	139.3 (6)	O12^ix^—K19—O16	89.80 (17)
C1—O11—K19^ii^	121.5 (6)	O12^ix^—K19—O17^xi^	125.68 (15)
K19—O11—K19^ii^	93.48 (13)	O12^ix^—K19—O21^xiii^	61.00 (17)
C1—O12—K19^iii^	100.4 (5)	O15^xii^—K19—O16	159.7 (2)
C1—O12—K19^iv^	106.9 (5)	O15^xii^—K19—O17^xi^	118.28 (14)
C1—O12—Li20^v^	148.3 (8)	O15^xii^—K19—O21^xiii^	117.64 (16)
K19^iii^—O12—K19^iv^	94.68 (12)	O16—K19—O17^xi^	70.13 (18)
K19^iii^—O12—Li20^v^	90.8 (7)	O16—K19—O21^xiii^	82.6 (2)
K19^iv^—O12—Li20^v^	101.5 (7)	O17^xi^—K19—O21^xiii^	66.53 (17)
C6—O15—K19^vi^	105.29 (16)	O12^xiv^—Li20—O12^ix^	125.0 (14)
C6—O15—K19^ii^	105.29 (16)	O12^xiv^—Li20—O15^xv^	114.1 (8)
C6—O15—Li20^vii^	153.2 (10)	O12^xiv^—Li20—O21^xiii^	97.2 (8)
K19^vi^—O15—K19^ii^	143.4 (2)	O12^ix^—Li20—O15^xv^	114.1 (8)
K19^vi^—O15—Li20^vii^	80.9 (3)	O12^ix^—Li20—O21^xiii^	97.2 (8)
K19^ii^—O15—Li20^vii^	80.9 (3)	O15^xv^—Li20—O21^xiii^	102.1 (11)
C6—O16—K19	127.43 (14)	K19^xvi^—O21—K19^ii^	85.1 (2)
C6—O16—K19^i^	127.43 (14)	K19^xvi^—O21—Li20^xvi^	94.2 (5)
K19—O16—K19^i^	104.7 (3)	K19^xvi^—O21—H22^xvii^	64.4 (7)
C3—O17—H18	93.0 (6)	K19^xvi^—O21—H22^ix^	132.6 (7)
C3—O17—K19^iii^	112.5 (4)	K19^ii^—O21—Li20^xvi^	94.2 (5)
C3—O17—K19^viii^	112.5 (4)	K19^ii^—O21—H22^xvii^	132.6 (7)
H18—O17—K19^iii^	129.8 (3)	K19^ii^—O21—H22^ix^	64.4 (7)
H18—O17—K19^viii^	129.8 (3)	Li20^xvi^—O21—H22^xvii^	55.8 (4)
K19^iii^—O17—K19^viii^	80.43 (16)	Li20^xvi^—O21—H22^ix^	55.8 (4)
O11—K19—O11^x^	158.80 (9)	H22^xvii^—O21—H22^ix^	110.0 (8)

Symmetry codes: (i) −*x*, *y*, *z*; (ii) −*x*+1/2, −*y*, *z*−1/2; (iii) *x*, *y*+1, *z*; (iv) −*x*+1/2, −*y*+1, *z*−1/2; (v) *x*+1/2, −*y*+1, *z*−1/2; (vi) *x*−1/2, −*y*, *z*−1/2; (vii) *x*, *y*, *z*−1; (viii) −*x*, *y*+1, *z*; (ix) −*x*+1/2, −*y*+1, *z*+1/2; (x) −*x*+1/2, −*y*, *z*+1/2; (xi) *x*, *y*−1, *z*; (xii) *x*+1/2, −*y*, *z*+1/2; (xiii) *x*−1/2, −*y*, *z*+1/2; (xiv) *x*−1/2, −*y*+1, *z*+1/2; (xv) *x*, *y*, *z*+1; (xvi) *x*+1/2, −*y*, *z*−1/2; (xvii) *x*+1/2, −*y*+1, *z*+1/2; (xviii) *x*−1/2, −*y*+1, *z*−1/2.

### (KADU1697_phase_2) Crystal data

**Table:**

C_6_H_5_KLi_2_O_7_	β = 80.6064°
*M**_r_* = 242.08	γ = 83.1095°
Triclinic, *P*1	*V* = 416.59 Å^3^
*a* = 6.48415 Å	*Z* = 2
*b* = 6.68334 Å	*D*_x_ = 1.930 Mg m^−^^3^
*c* = 9.81709 Å	*T* = 300 K
α = 87.6373°	

### (KADU1697_phase_2) Fractional atomic coordinates and isotropic or equivalent isotropic displacement parameters (Å^2^)

**Table:**

	*x*	*y*	*z*	*U*_iso_*/*U*_eq_	
C1	−0.15531	0.06638	0.32685	0.05983*	
C2	−0.02851	0.23221	0.35291	0.02275*	
C3	0.1869	0.25126	0.26327	0.02275*	
C4	0.24628	0.42934	0.33775	0.02275*	
C5	0.4347	0.52818	0.26817	0.05983*	
C6	0.118	0.34833	0.12921	0.05983*	
H7	0.00819	0.21576	0.46276	0.02058*	
H8	−0.13090	0.38187	0.34466	0.02958*	
H9	0.28351	0.37630	0.44379	0.02958*	
H10	0.10450	0.54936	0.35301	0.02958*	
O11	−0.30929	0.02453	0.41739	0.05983*	
O12	−0.10754	−0.03339	0.21622	0.05983*	
O13	0.53582	0.66706	0.29883	0.05983*	
O14	0.54742	0.43056	0.16909	0.05983*	
O15	0.00612	0.52016	0.14052	0.05983*	
O16	0.2083	0.21459	0.0417	0.05983*	
O17	0.32695	0.07281	0.22514	0.05983*	
H18	0.394	0.1785	0.2331	0.06878*	
K19	0.74052	0.18609	−0.02971	0.04605*	
Li20	0.74351	0.74584	0.16276	0.05*	
Li21	0.55124	0.10113	0.63762	0.05*	

### (KADU1697_phase_2) Geometric parameters (Å, º)

**Table:**

C1—C2	1.5101	O15—K19^i^	3.5935
C1—O11	1.2736	O15—K19^v^	2.7841
C1—O12	1.2730	O15—Li20^i^	2.1241
C2—C1	1.5101	O16—C6	1.2859
C2—C3	1.5407	O16—K19^i^	3.2514
C3—C2	1.5407	O16—K19	3.3912
C3—C4	1.5404	O16—K19^iii^	2.6637
C3—C6	1.5507	O16—Li20^v^	1.9919
C3—O17	1.4328	O17—C3	1.4328
C4—C3	1.5404	O17—K19	3.4878
C4—C5	1.5099	O17—K19^iii^	2.7561
C5—C4	1.5099	O17—Li21^vii^	1.9464
C5—O13	1.2711	K19—O12^viii^	3.0147
C5—O14	1.2695	K19—O12^iii^	2.8647
C6—C3	1.5507	K19—O13^v^	3.4733
C6—O15	1.2813	K19—O14	2.6496
C6—O16	1.2859	K19—O14^v^	3.3644
O11—C1	1.2736	K19—O15^viii^	3.5935
O11—Li21^i^	2.2575	K19—O15^v^	2.7841
O11—Li21^ii^	2.0220	K19—O16	3.3912
O12—C1	1.2730	K19—O16^viii^	3.2514
O12—K19^i^	3.0147	K19—O16^iii^	2.6637
O12—K19^iii^	2.8647	K19—O17	3.4878
O12—Li20^iv^	1.9891	K19—O17^iii^	2.7561
O13—C5	1.2711	Li20—O12^ix^	1.9891
O13—K19^v^	3.4733	Li20—O13	1.8439
O13—Li20	1.8439	Li20—O14	2.582
O13—Li21^vi^	1.6920	Li20—O15^viii^	2.1241
O14—C5	1.2695	Li20—O16^v^	1.9919
O14—O13	2.0573	Li21—O11^viii^	2.2575
O14—K19	2.6496	Li21—O11^ii^	2.0220
O14—K19^v^	3.3644	Li21—O13^vi^	1.6920
O14—Li20	2.582	Li21—O17^vii^	1.9464
O15—C6	1.2813		
			
C2—C1—O11	119.5504	C3—O17—H18	67.1832
C2—C1—O12	120.5205	C3—O17—K19^iii^	122.505
O11—C1—O12	119.9213	C3—O17—Li21^vii^	121.6382
C1—C2—C3	120.0207	H18—O17—K19^iii^	140.8897
C2—C3—C4	97.8299	H18—O17—Li21^vii^	97.505
C2—C3—C6	100.9126	K19^iii^—O17—Li21^vii^	104.7899
C2—C3—O17	119.4522	O12^viii^—K19—O12^iii^	93.0913
C4—C3—C6	103.978	O12^viii^—K19—O14	80.1974
C4—C3—O17	124.1292	O12^viii^—K19—O15^v^	112.4929
C6—C3—O17	107.4222	O12^viii^—K19—O16^viii^	58.136
C3—C4—C5	116.8773	O12^viii^—K19—O16^iii^	64.5695
C4—C5—O13	135.3652	O12^viii^—K19—O17^iii^	112.5733
C4—C5—O14	114.8354	O12^iii^—K19—O14	151.5549
O13—C5—O14	108.1461	O12^iii^—K19—O15^v^	66.0201
C3—C6—O15	116.7212	O12^iii^—K19—O16^viii^	59.37
C3—C6—O16	99.9634	O12^iii^—K19—O16^iii^	66.8873
O15—C6—O16	143.2807	O12^iii^—K19—O17^iii^	64.5387
C1—O11—Li21^i^	138.7284	O14—K19—O15^v^	90.893
C1—O11—Li21^ii^	120.5833	O14—K19—O16^viii^	94.4416
Li21^i^—O11—Li21^ii^	99.3848	O14—K19—O16^iii^	131.1798
C1—O12—K19^i^	113.4217	O14—K19—O17^iii^	143.4371
C1—O12—K19^iii^	139.176	O15^v^—K19—O16^viii^	56.1366
C1—O12—Li20^iv^	126.3908	O15^v^—K19—O16^iii^	132.5368
K19^i^—O12—K19^iii^	86.9087	O15^v^—K19—O17^iii^	112.9108
K19^i^—O12—Li20^iv^	83.8856	O16^viii^—K19—O16^iii^	94.3136
K19^iii^—O12—Li20^iv^	89.1605	O16^viii^—K19—O17^iii^	121.6786
C5—O13—Li20	116.3317	O16^iii^—K19—O17^iii^	48.0157
C5—O13—Li21^vi^	130.5031	O12^ix^—Li20—O13	114.3245
Li20—O13—Li21^vi^	96.8988	O12^ix^—Li20—O15^viii^	96.8417
C5—O14—K19	171.2327	O12^ix^—Li20—O16^v^	99.9031
C6—O15—K19^v^	108.1884	O13—Li20—O15^viii^	109.5957
C6—O15—Li20^i^	161.8601	O13—Li20—O16^v^	138.1587
K19^v^—O15—Li20^i^	88.7083	O15^viii^—Li20—O16^v^	88.3426
C6—O16—K19^i^	85.8733	O11^viii^—Li21—O11^ii^	80.6152
C6—O16—K19^iii^	137.0461	O11^viii^—Li21—O13^vi^	127.2048
C6—O16—Li20^v^	125.4341	O11^viii^—Li21—O17^vii^	113.934
K19^i^—O16—K19^iii^	85.6864	O11^ii^—Li21—O13^vi^	109.7009
K19^i^—O16—Li20^v^	78.6777	O11^ii^—Li21—O17^vii^	109.0825
K19^iii^—O16—Li20^v^	93.8091	O13^vi^—Li21—O17^vii^	110.782

Symmetry codes: (i) *x*−1, *y*, *z*; (ii) −*x*, −*y*, −*z*+1; (iii) −*x*+1, −*y*, −*z*; (iv) *x*−1, *y*−1, *z*; (v) −*x*+1, −*y*+1, −*z*; (vi) −*x*+1, −*y*+1, −*z*+1; (vii) −*x*+1, −*y*, −*z*+1; (viii) *x*+1, *y*, *z*; (ix) *x*+1, *y*+1, *z*.

### (kadu1697_DFT) Crystal data

**Table:**

C_6_H_7_K_2_LiO_8_	*b* = 5.8658 Å
*M**_r_* = 292.25	*c* = 8.1929 Å
Orthorhombic, *P**m**n*2_1_	*V* = 492.53 Å^3^
Hall symbol: P 2ac -2	*Z* = 2
*a* = 10.2488 Å	

### (kadu1697_DFT) Data collection

**Table:**

*h* = →	*l* = →
*k* = →	

### (kadu1697_DFT) Fractional atomic coordinates and isotropic or equivalent isotropic displacement parameters (Å^2^)

**Table:**

	*x*	*y*	*z*	*U*_iso_*/*U*_eq_	
C1	0.25085	0.55039	0.91138	0.01550*	
C2	0.12106	0.61656	0.83349	0.01010*	
H7	0.12406	0.56332	0.70555	0.01310*	
H8	0.10941	0.80147	0.83534	0.01310*	
O11	0.27114	0.34238	0.94262	0.01550*	
O12	0.33128	0.71110	0.93752	0.01550*	
K19	0.19597	−0.04778	1.18244	0.03330*	
H22	0.42322	0.21715	0.95731	0.04000*	
C3	0.00000	0.51342	0.91548	0.01010*	
C6	0.00000	0.25095	0.89891	0.01550*	
O15	0.00000	0.16665	0.75711	0.01550*	
O16	0.00000	0.14035	1.03069	0.01550*	
O17	0.00000	0.57368	1.08543	0.01550*	
H18	0.00000	0.42546	1.14047	0.02010*	
Li20	0.00000	0.19424	0.52218	0.04000*	
O21	0.50000	0.11887	0.94174	0.01800*	

### (kadu1697_DFT) Bond lengths (Å)

**Table:**

C1—C2	1.525	C3—C6	1.546
C1—O11	1.264	C3—O17	1.437
C1—O12	1.270	C6—O15	1.263
C2—C3	1.535	C6—O16	1.260
C2—H7	1.094	O15—Li20	1.932
C2—H8	1.091	O16—K19^vii^	2.607
O11—K19^i^	2.765	O17—H18	0.979
O12—K19^ii^	2.889	O17—K19^iii^	3.098
O12—K19^iii^	2.820	O17—K19^viii^	3.098
O12—Li20^iv^	1.944	Li20—O12^ii^	1.944
K19—O12^iv^	2.889	Li20—O12^ix^	1.944
K19—O16	2.607	Li20—O21^i^	1.951
K19—O11^v^	2.765	O21—Li20^v^	1.951
K19—O17^vi^	3.098	O21—K19^i^	2.953
K19—O12^vi^	2.820	O21—K19^x^	2.953
K19—O21^v^	2.953	O21—H22	0.984
H22—O21^ii^	0.984	O21—H22^xi^	0.984
C3—C2^vii^	1.535		

Symmetry codes: (i) −*x*+1/2, −*y*, *z*−1/2; (ii) −*x*+1/2, −*y*+1, *z*−1/2; (iii) *x*, *y*+1, *z*; (iv) −*x*+1/2, −*y*+1, *z*+1/2; (v) −*x*+1/2, −*y*, *z*+1/2; (vi) *x*, *y*−1, *z*; (vii) −*x*, *y*, *z*; (viii) −*x*, *y*+1, *z*; (ix) *x*−1/2, −*y*+1, *z*−1/2; (x) *x*+1/2, −*y*, *z*−1/2; (xi) −*x*+1, *y*, *z*.

### (kadu1697_DFT) Hydrogen-bond geometry (Å, º)

**Table:**

*D*—H···*A*	*D*—H	H···*A*	*D*···*A*	*D*—H···*A*
O21—H22···O11	0.98	1.73	2.687	164
O17—H18···O16	0.98	1.90	2.581	124
C2—H7···O11^ii^	1.09	2.47	3.396	142

Symmetry code: (ii) −*x*+1/2, −*y*+1, *z*−1/2.
